# The British Red Cross and Order of St. John Come to the Help of the Voluntary Hospitals

**Published:** 1920-01-31

**Authors:** 


### BRITISH HOSPITALS ASSOCIATION.

### The British Red Cross and Order of St. John come to the Help of the Voluntary Hospitals.

A largely-attended and representative meeting of hospital governors and secretaries of the country was held, under the auspices of the British Hospitals Association, on Friday, January 23, in the Great Hall of St. Thomas's Hospital, to hear and discuss the proposal for co-operative working with the Red Cross agencies which Sir Arthur Stanley had undertaken to submit.

In the regretted absence of Lord Sandhurst, the chair was occupied by Mr. H. Wade Deacon, of the Liverpool Royal Infirmary, and among those supporting him were Lord Knutsford, Sir Arthur Stanley, G.B.E., Sir Henry Burdett, K.C.B., Mr. Leo Lyle, M.P., Sir Robert Morant (representing the Minister of Health) Sir Napier Burnett, M.D., Col. D. J. Mackintosh (Western Infirmary, Glasgow), Major Rhodes (Ministry of Pensions), Rev. G. B. Cronshaw (Radcliffe Infirmary, Oxford), Mr. F. G. Hazell (Manchester), Mr. R. H. P. Orde (Newcastle-on-Tyne), and Mr. C. S. Risbee (Northampton).

There were also present: Rev. B. W. Bradford (Banbury), Lieut.-Col. F. Ashley (British Red Cross), Dr. H. Willingham Gell (Reading), Monsignor M. E. Carton de Wiart (St. Andrew's Hospital), Sir J. Lynn Thomas (Cardiff), Mr. W. McAdam Eccles, F.R.C.S. (St. Bartholomew's Hospital), Hon. R. C. Parsons, Sir Courtauld Thomson (Order of St. John), Sir James Harrison (National Hospital for Heart), Sir M. D. Chalmers, K.C.B. (London Fever Hospital), Col. J. E. Broadbent (Reading), Rt. Hon. Evelyn Cecil (Order of St. John), Sir R. Fox Symons, K.B.E. (B.R.C.S.), Sir R. C. Temple, and Messrs. Alfred Langton (Royal Free Hospital), Arthur Preston (Guildford), James Churchfield (St. George's), G. Cunningham (Middlesex), G. F. Sheppard (Royal Free), Arthur Moore (Lincoln), G. H. Hamilton (National, Queen Square), Arthur Lucas (Hospital for Sick Children), C. P. Lovelock (Carshalton), W. H. Head (Cambridge), Thomas Penty (York), W. Deacon (Bridgwater), Fredk. Gore (Margate), Fredk. Neden (York), B. V. Melville (Royal Free), E. Victor Dodd (Coventry), Arthur Watts (Queen Charlotte's), S. Clayton Fryers (Sunderland), Fredk. Drewett (Prince of Wales General), R. G. Garrett (Royal Free), George H. Dams (Croydon), H. R. Maynard (King Edward's Hospital Fund), O. P. Maddick (Wellingborough), F. Brown (Folkestone), F. J. Hancock (West Bromwich), R. W. Reid (Folkestone), P. M. MacColl (Nottingham), T. B. Harrison (Buxton), Walter Banks (Derby), R. Fielding (Blackpool), L. W. Lancaster-Gaye (Brighton), G. A. Arnaudin (St. John's Skin), Charles Irwin (Newcastle-on-Tyne), F. Smallpeice (Guildford), James Phillips (Bradford), W. J. Morton (Mount Vernon), Henry A. Bone, and John East (Miller, Greenwich), D. L. Rutherford (Barrow-in-Furness), Woolmer White (Portsmouth), H. W. C. Drury (Norwich), John H. Robinson (Miller, Greenwich), William Sandover (Richmond), R. J. E. Whitney (National for the Heart), W. Vaux Graham (Westminster), W. J. Wormell (Coventry), H. Burleigh (Hospital for Epilepsy), R. H. Caird (London Homœopathic), P. B. Burgoyne and Chas. W. Cox (Ventnor), Herbert Lewis (Cardiff), W. H. Harper (Wolverhampton), A. H. Leaney (Birmingham), Timothy Warren (Glasgow), C. R. W. Offen (Leamington), G. Sunnuck (Chesterfield), Gilbert D. Shepherd (Cardiff), Allen Naldrett (Liverpool), Gordon Richards (Seaford), Alexander Pym (Royal Waterloo Hospital), A. Griffiths (Ipswich), Archibald D. Reid, B. Wagstaff (Portsmouth), H. H. Jennings (Chelsea for Women), J. H. Cooke (Winsford), J. W. Barnes (Sheffield), S. M. Quennell (Westminster Hospital), R. J. Bland (Royal London Ophthalmic), E. A. Humphries (Bradford), R. Hilder (Nurses' Co-operation), Edward Leech (Blackpool), F. Stanley Cheer (Paddington Green), D. Wells Patterson (Newcastle-on-Tyne), F. Hatch (Stoke-on-Trent), W. Stevenson (Stoke-on-Trent), A. G. Buck (Colchester), Gordon Saul (Bournemouth), W. J. Patrick (Guildford), C. C. Osborne (St. Leonards), Frank Inch (Norwich), Robert Gurney (Norwich), C. E. Allan and H. G. Evered (Victoria for Children, Chelsea), H. J. Eason (London Lock), F. G. Gardiner (Kent and Canterbury), George Watts (City of London Chest), G. Panter (Great Northern, Holloway), Thomas Ryan (St. Mary's), F. Wood (Brompton), G. P. Pemberton (B.R.C.S.), George F. Furness (Passmore Edwards, Willesden), Chas. T. Walrond (Hospital for Sick Children), W. W. Bryant (Cheltenham), Harold Wigg (Hampstead), Edwin J. Pearce (Stockport), J. Courtney Buchanan (Hon. Secretary), and R. A. Owthwaite (Hon. Treasurer).

**The Chairman** said all in the room were aware that the voluntary hospitals of the country were approaching a time of acute crisis; many were already spending more than they were receiving, some very much more, and there seemed but little prospect of relief until it was known what would be the condition of affairs when the full activities of the Ministry of Health had developed. At present there was no system for connecting or co-ordinating hospitals with one another. The scheme which had been published in the *Times* might be all very well for the not immediate future; what was urgently required was money with which to carry on until such schemes could be elaborated. To-day the meeting was favoured by the presence of Sir Arthur Stanley, who had made a definite suggestion to come to their assistance: it did not embrace any idea of either management or control. It was a voluntary offer from voluntary organisations to help the voluntary hospitals; all the hospitals were required to do was to keep their accounts in a certain form. There was no obligation upon any hospital to accept the offer. Most, however, would feel only too thankful to anybody who could help them, and would give a hearty welcome to Sir Arthur Stanley. At a meeting last Wednesday of the Medical Charities of Liverpool this scheme was discussed, and it was given a most cordial welcome, and he had been authorised by that meeting to come here to-day and strongly support any similar resolution which might be proposed. He felt that the meeting would be with him in this.

**Sir Arthur Stanley** said he was very glad he had had the opportunity of listening to the Chairman's opening remarks, because he had placed the matter before the meeting in exactly the right light. All he wished to say in comment was, that if he knew of any method by which money could be conjured up for the support of the voluntary hospitals, he would ask for a good share for his own hospital (St. Thomas's) before passing it on—(laughter). Schemes must be elaborated, and must of necessity take a little time before they were operative. Before coming to a description of the scheme itself, there were two remarks he wished to make. In the little pamphlet he had sent out he made allusion to the League of Mercy in a way which might lead to it being inferred that the work of that League was only for the London hospitals. That was no longer so, for its operations had now been extended to other than the immediate home counties, and to other parts of the Dominions, and he would be sorry if anything he wrote might appear to be a disparagement of their efforts. On the contrary, it was by the combination of these voluntary associations that it was hoped to help the hospitals. The other point was, that whenever he made allusion to the Red Cross, he included in the term the Order of St. John: that was agreed by representatives of both bodies.

The scheme he had to put to the meeting—which was, in fact, a mere outline—had already been sent to representatives of the Association, and the case was excellently put in the article in that day's *Times* by Mr. Mayhew. At the very outset he wished to make it clear that in this suggestion the Red Cross were not in any way trying to infringe upon any of the duties or rights that ought to be, and continued to be, exercised by the State or by the local authorities. We now had a Ministry of Health, for which he felt most heartily grateful, because it meant an immense benefit to the country. That Ministry had got very definite duties to perform to the State, and he was convinced those duties would be carried out. But there was always scope for voluntary effort beyond this, and it was just that voluntary effort it was desired to bring to bear in the present instance. It was being largely exercised in voluntary hospitals. The Red Cross possessed a big organisation, and they wanted to see whether it could effectively come to the aid of the voluntary hospitals of the country. In fact, the Red Cross wished to stand, in time of peace, in connection with hospitals and with work generally, in the same relation to the Ministry of Health as during the war it stood to the War Office. It was not for the Red Cross to say what policy should be pursued, it was not for them to attempt to interfere with State prerogatives, but to bring to the help of the State everything they could. Dr. Addison, it was hoped, might have been able to attend this meeting, but he had been prevented by his Ministerial duties; still he was glad to see

**British Hospitals Association**—(*continued*).

present Sir Robert Morant, the representative of the voluntary hospitals throughout the country, and they had no more sympathetic friend. There was one thing that all at this meeting, at all events, would be agreed upon, namely, that if it could possibly be done, the voluntary hospital system in this country must be maintained. And he thought his hearers would also agree with him that the public of this country, who during the war had learned to give generously, would now, since the war, find the money for these voluntary hospitals, provided the machinery of collection was supplied, and if they could be well satisfied that the money they might give would be put to the best use. Hospitals had been built up and maintained in the past by the munificence and benefactions of a particular class, and it was that class which had been most hard hit during the war, the people with very large fixed incomes. Taxation and the cost of living had hit them so hard that they could not now respond in the same degree as they had in the past. On the other hand, there was another class—numerically by far the largest class—which had benefited, he would not say by the war, but whose position after the war was undoubtedly better than it was before the war; he referred to the vast working-class community of this country. He had had a certain amount to do with collecting money during the last five years, and he said, without fear of contradiction, that the big community in this country to which he had referred were just as willing and ready to give as were the old class of givers, if only there could be effectively brought to their notice the necessity of giving, and the machinery by which the money could be obtained. He did not think there was anything to be gained by having a great collection-day for hospitals, such as had been raised for the Red Cross. Even when all the excitement and incitement of the war was on, in the last year of it, when people had learned to give freely, and when they were inclined to be more than generous with the Red Cross, with all the Red Cross organisation at full strength, "Our Day" yielded in collection only £3,750,000, and he believed that at least half of that came from overseas. He did not wish to speak slightingly of a sum like that; if the hospitals could get such a sum they would be very glad; but if in a time of war that was all that an organised collection of the kind could do, one could not look for anything very enormous from a day collection in peace time.

Now the war was over he did not know how far the Red Cross organisation would be held together; and one came to this: The only way in which to form an opinion on the value of an organisation was to put figures forward, which in this instance must be regarded as somewhat fanciful, though some of them would be found to be pretty correct. In this country at the present time there were 300 hospitals, with an average of 150 beds each, a total bed accommodation of 45,000. At this date the upkeep of such beds could be put at £150 per annum each, which meant that every year there was a need to raise £6,750,000. He had consulted with his friend, Sir Henry Burdett, who was the greatest authority in the country on these matters; and he told him (the speaker) it would not be safe to conclude, on the average, that these hospitals were more than one-third endowed: from their investments they could not be sure of getting more than one-third.* Deducting one-third from the 6$\frac{3}{4}$ millions, there remained about £4,000,000 to be raised, and the question was as to how that was to be done in small sums. What he was about to say was purely a counsel of perfection which might not be attained. Assuming the population of this country to be about 40 millions, surely there were at least a million people who, out of their wages, could afford to put by a shilling per week for the hospital. If they did, it would produce a revenue of about £2,500,000 a year. Surely, also, there were two million people who could each afford sixpence a week for the hospital, which would produce a like sum, totalling more than the sum required. And that touched only about 9 per cent. of the population. At the present time less than 10 per cent. of the people subscribed to hospitals. If there were—as he believed they actually possessed—an organisation which extended right through the country and had in it an immense amount of local patriotism, he could not believe it would be found to be an impossible task to raise every year the required four million pounds for the hospitals. That gave a general idea of the way in which, he thought, the Red Cross organisation would be able to help most. But the people giving the money wanted to be sure, in their own minds, that the money given by them would be put to the best use. That assurance could only be given if they were shown that every possible step was taken to secure efficiency and economy in their own hospital. When the Red Cross had 1,600 or 1,700 auxiliary hospitals under its care, they found, when they began to get the figures, extraordinary divergencies in the matter of expense in running them; and most people experienced in the running of hospitals would agree that it was of the greatest possible use to issue statistics and comparative tables. Lord Knutsford did not always agree with comparative tables, and he (Sir Arthur) was willing to admit that too much could be made of them. Still, if, by a study of the statistics, a hospital board found it was spending either very much below or very much above the average, it was useful for it to know the fact and to look into its own accounts. One of the interesting things they found during the war was that as many hospitals were spending too little—were not giving sufficient and proper food—as were spending too much. In order that the public could be assured that every care would be used to promote economy and efficiency, it was necessary there should be a recognised expert. Such an expert, he was very glad to be able to announce, they had been able to find in the person of Sir Napier Burnett, a well-known expert, who did more to promote economy and efficiency than any other man, and who would be able to devote the whole of his time to the work. He was also grateful to Mr. Herman Darewski, who did an immense amount of work in connection with the Red Cross and many other charitable organisations, for having made the appointment of Sir Napier Burnett to the post possible. Whatever was done, they must depend on the earnest support of the general public. In the early days of the Red Cross the collectors handed in exactly £7,000, and their expenditure immediately went far beyond their means. Those who were responsible for the finances of the Red Cross scolded him on many occasions, and told him he was going too far. The only answer he was able to give was that if the work did not commend itself to the public it would very soon stop from want of funds; but that if they did do what was helpful, if the public did get confidence in the organisation, they would provide for all its needs. His hearers now knew how splendidly the Red Cross appeal was responded to during the war, for during those five years they were able to raise about £19,000,000. If in the present matter a good case could be presented to the public, then, if the public felt they were going to get good value for their money, that what they would give to the hospitals would be returned, and more than returned, by the saving of the life of the nation by the improvement in the general standard of health, he looked forward to being able to bring about, for this great object, some of that general support which had been given by the public during the war.

**Lord Knutsford** moved the following resolution:

"That the thanks of the British Hospitals Association be conveyed to the Joint Council of the British Red Cross Society and Order of St. John for their offer of assistance, which is cordially and gratefully accepted by the hospitals represented at this meeting."

He said it was really extremely unselfish of him to do so, because he understood that this aid which the Red Cross hoped to give to hospitals was not to apply to the London ones. As, however, unselfishness had always been one of his qualities, he moved the resolution without further comment. (Laughter.)

**Rev. G. B. Cronshaw** seconded, expressing his hope for the success of the project.

**Mr. R. H. P. Orde** (Newcastle) said he took it the representatives of the hospitals had been invited to the meeting so that they could express their opinions on Sir Arthur Stanley's proposal, so that an idea might be formed as to its possible reception throughout the country. Probably no body in the country was more capable of giving such a line than was this Association. Provincial hospitals were accustomed to rely largely on local support for the upkeep of their hospitals, but probably no area in England tapped all its available resources. In every area there were what might be called "cranky" donors, men and women who would far rather support a hospital in some other area than one in their own; and each hospital tapping its own residents, the donations of cranky ones did not get to any hospital. There was also a large class of rich persons and firms who were inundated with appeals, but disliked discriminating between the merits of the different applicants. Such would welcome the Red Cross making up their minds for them. He did not think any body would be more suitable or acceptable for the proposed work than the Red Cross; its history and its war record had secured the confidence of the whole country. He sincerely hoped the scheme would be a success, for it would mean the hospitals were to be helped by a body with aims and aspirations similar to their own; one which, while it asked agreement to certain reasonable standards and conditions, would still leave to the hospitals their individuality and their power to develop along their own lines. One of the points in the scheme was that the Red Cross should establish a central bureau, where information on matters of hospital interest would be available. Each hospital had its own excellences, and although the hospitals were only too ready to impart their experiences, yet one hesitated to trespass on kindness when it would involve the hospital in a very considerable amount of trouble. His hospital had no means of sharing the bureau which Sir Arthur Stanley men-

* Taking 106 general hospitals dealt with in the Income and Expenditure tables of Burdett's "Hospitals and Charities" for 1919 (see idem, p. 140), the percentage of invested property to total ordinary income in the year 1917 is as follows:

| | |
|---|---|
| 26 London General Hospitals ... | ... 30.8 |
| 65 Provincial General Hospitals | ... 15.2 |
| 11 Scottish General Hospitals ... | ... 23.9 |
| 4 Irish General Hospitals ... | ... 17.5 |

ED. *T. H.*]

**British Hospitals Association—(*continued*).**

tioned in his pamphlet. The art of raising money had now reached a very high pitch of excellence, but the art of saving money had been comparatively neglected. By that he did not mean beggarly economies on food, salaries, drugs, and dressings; but savings which could be effected by a timely expenditure on capital. He had often thought there ought to be some authority in the country from which hospitals could borrow money, at a reasonable rate of interest, on approved schemes. He did not know whether that came within the purview of Sir Arthur Stanley's scheme, but it was worth considering. Such schemes could only be undertaken on the most perfect information, such information as only a bureau of the kind mentioned could supply.

A Surrey representative asked how it was proposed to collect the money. He was glad to hear the assurance that nothing in the way of management of local hospitals by the Red Cross was intended. His own colleagues had a suspicion that control would be attempted by the county areas, and the prospect was not liked. He wanted an assurance that the outcome of the scheme would be pooled with very great care.

**Mr. Humphries** (Bradford) said the papers would have told hearers that Bradford had a lot of money. There was a lot of money at Bradford, and a lot to be collected. He knew of large sums ready to be given to hospitals—which he thought would be given to the Red Cross—if the intended donors could have some information as to what the State and the municipal hospitals were going to do. In his town there was a hospital which had been started by the Municipality with 1,200 beds, and donors who had thousands of pounds to give would not hand it over until they had a definite assurance that the hospitals were not going to be taken over by the State. At a suitable time he intended to move a resolution asking that the Ministry of Health should co-operate with this meeting to formulate a plan of approaching intending subscribers. Money was urgently wanted; the hospitals were starving for it.

**Sir Henry Burdett, K.C.B.,** expressed his agreement with the last speaker. It had been his own experience in all parts of the country, and from all kinds of institutions, that this was the case. Questions had been asked in Parliament of the Minister of Health, and, in substance, the answers were that the Government was not going to nationalise the hospitals or take them over. It intended to leave them alone. No doubt Sir Robert Morant would give this meeting a similar assurance. Our difficulty at the present day was this—and he had been getting out and analysing the audited returns of the chief hospitals of the country. They got on extremely well, and had a good surplus from 700 to 800 hospitals, up to and including 1917. The trouble with them began when the war got thicker, expenses were heavier, and wounded were arriving in greater numbers, the whole call on the hospitals being very much increased. That was still the case in the next year, and there still remained the years ahead. The voluntary hospitals did not know, at the present moment, where they were financially. There were still three years to go through, years which must be difficult owing to war conditions and their adjustment. He believed that readjustment, in the ordinary calculations of business men, should be settled within three years. There was thus a three-year period for the hospitals to work in, and while they had to thank Sir Arthur Stanley and the Red Cross from their hearts for this spontaneous and voluntary offer of most material service—material in two directions, the money it was hoped to raise and the assistance of willing workers who would come to the aid of the voluntary hospitals in every large community in England, and Scotland, too, he hoped—yet this financial business could not be got straight without the personal exertions of those concerned. These would be handicapped, however, because those who had supported the hospitals heretofore, though few relatively to the whole population, were, many of them, severely hit by the war, so that they would be unable to go on giving the sums which they had contributed to the hospitals in the past. But there remained a very large section yet to be appealed to. Making due allowance for children and old people, he considered there were 20 million people in Great Britain who probably had never done anything for, or given anything to, hospitals. In studying the question and going among the people in the days when he was preaching personal service—which he had been doing for decades of years—he found that people were really willing. Among them were some of the most important and zealous workers, and the need was to try and get them interested in this work. The days for sentimental talk and for applying the word "charity" to our great voluntary hospitals had now passed. It was not a question of charity at all, but rather one of self-defence and humanity. No single person, whether rich or poor, could do without the hospital when ill, and it was necessary to get that fact recognised throughout the land. There should be provision in our hospitals for all classes, and that should not be very difficult to bring about. At least once a week he received a request for advice as to the provision of further accommodation of various kinds. The wish was there, as was also the power to give, because, as Sir Arthur Stanley had said, there were large numbers of the community who had made heavy profits, and he (Sir Henry) believed those people had now got over the excitement of spending and had arrived at the stage at which the spending of their money was a bore. If they could be approached at that stage, it would be found that they were quite willing to give to hospitals. He had had experience of that, for during fifteen years no less than 3½ million pounds had been paid into his own letter-box for hospitals. He had given up that work now because experience has taught him that one man could appear too often as a raiser of funds. And there might be merit in changing the personal channel of the gift; people probably got tired of giving to institutions always through the same persons. If so, those persons should be induced to take a back seat. This was an important point to bear in mind. He urged his hearers to put their minds into the matter, to influence their associates and their local Press in furtherance of this work. Three years remained in which to get the money, and he believed it could be done. The people should combine with earnest purpose to give the nation an opportunity of putting hospital finances everywhere on an adequate basis by the co-operation of men and women of all classes throughout the land. Since his scheme to accomplish this was published in the *Times* on December 17, community after community had been awakened and had got to work, and the results every week were proving so encouraging as to be in some instances, as in that of Cardiff, simply magnificent. The Lord Mayor of that City, a man of great energy and vigour, dropped all questions of charity and sentiment and appealed to his people on the broad ground of humanity. He asked for £150,000, and in less than 100 hours half the money had been received. The outlook for the London hospitals was much graver in 1896, but with the aid of the King's Hospital Fund and the faith and untiring zeal of the lamented King Edward and those who gathered round him, the crisis was successfully surmounted. To-day we had the war crisis for the voluntary hospitals all over the country, which also, he felt confident, could be met by united action and wise policy, and by the co-operation of the majority of the population everywhere. The progress already made proved that the people were willing and anxious to give themselves and their money for this great object if they were properly approached and made thoroughly acquainted with the requirements. This week he had tried to show how this could be done in every locality, and particulars would be found in The Hospital of how this had brought wonderful results in the last six or eight weeks in the communities which had resolutely taken the matter in hand. In each locality a live man to lead the movement, with some good organisers, was essential. and then he felt certain that all the money shown to be needed would be forthcoming.

**Mr. Jennings** (Chelsea Hospital for Women) did not think London was very fortunate in this proposed scheme. Was it likely that people would take part in helping the various war depôts which had been set up if the assistance proposed was to leave out London? He thought the door should be thrown open to London.

**Sir Robert Morant** said the Minister of Health himself had been anxious to be present, but had been prevented by other engagements. Dr. Addison wished this meeting definitely to understand what he had already declared on three separate occasions in the House of Commons—that there was no foundation for statements which had been rife of late to the effect that the establishment of a Ministry of Health was intended to mean the imposition on the voluntary hospitals of bureaucratic control. There was no such intention. On the contrary, this being a meeting, as the Minister understood, for furthering and supporting the voluntary effort, not only, he hoped, to do what it had been doing, but to extend its purview and diminish the waiting-lists and increase the possibilities, Dr. Addison was anxious it should be known to the meeting that he regarded the efforts with the utmost sympathy and interest, and would hope in every conceivable way to foster co-operation, so far as that was desirable, between the Ministry on the one hand and such organisation as this meeting might bring into existence, with Sir Napier Burnett, on the other. Therefore the Minister was virtually here to say he was keen on this movement, was anxious to see it successful, and would co-operate with it in every possible way.

**Sir Lynn Thomas** (Cardiff), speaking on behalf of a small Cardiff hospital for soldiers and sailors who lost limbs in the war, said a census in Wales showed that these were outnumbered by civilians who were similarly crippled. He mentioned the matter because the effort was a very big one, and he felt sure more money would be required than had been named.

Another Welsh representative confirmed what Sir Henry Burdett said as to the special effort in Cardiff, and added that £40,000 was being appealed for in order to secure an annual income. The Red Cross organisations in Wales had banded themselves together and agreed to support the scheme submitted by Sir Arthur Stanley to-day. Large numbers of workers in Wales would agree to give a penny a week to the hospitals for ever. If that could be done throughout the country there would be no difficulty in getting the needed money.

**British Hospitals Association**—(*continued*).

**Sir Napier Burnett, K.B.E., M.D.,** said he would like to say a few words in regard to some of the questions which had been raised. This question had a fascination for him, because he knew that the central idea in the outstanding problem now under discussion was help—help to the individual hospitals, and, from that, help to the country at large. There was to be no interference with the individuality of any hospital, nor with the effort put forth by any hospital. The efforts which would arise from this scheme, if it should receive the meeting's blessing, were simply in supplement of those of the individual hospitals. It would not only give suggestions as to how to raise money, but it would hope also actually to give the cash. It would also be delighted to give to hospitals instructions as to how to raise their own funds. And, in regard to any interference with the collecting activities of other agencies, he wished to make this point very clear. The Red Cross, in its collecting of money in "Our Day," would in no sense collide with the local efforts made by any hospital or other fund. Before the Red Cross came to any district to collect money in its own way, it would communicate with all hospital authorities to see if they would prefer they stayed out. That would answer the speaker from that Eldorado, Bradford; and he, the speaker, would give the Bradford representative heart to go back and begin tapping those mines of wealth to-morrow. And the Red Cross would take Sir Henry Burdett's hint and appeal to the boredom of those who had tired of spending. One representative spoke on behalf of London hospitals. Poor London! (Laughter.) He (the speaker) came from a poor country—(Laughter)—which generally had its eye on London; but, though London was a big place, it was not England. Moreover, London had its King's Fund. The provincial hospitals had nothing, and this was a first step towards helping the provincial hospitals, to some of which important medical schools were attached, and an attempt would be made to help them along the same lines as the King's Fund had done for London. Yesterday, he agreed to act as Director of the Hospital Services of this movement. He knew the money would be forthcoming, and he hoped to make some distribution before the close of the present financial year. He appealed for all the help which could be given. They would stand being criticised, and in return would criticise. There was nothing destructive about the scheme; they wanted to preserve these glorious institutions. The description given to them by a famous American was true: they were "excellent hostelries for the sick"; but he suggested that the hostels of this country might do a little more than they were doing at present; they were capable of a fuller service than they had yet supplied. By some co-operative effort the excellences of one district could be brought to another, and by a general appeal the standard of the whole could be raised. There were other functions connected with a hospital besides treating the sick—namely, those of teaching and research, and the need for these he eloquently emphasised.

**Mr. Leo Lyle, M.P.,** referred to the question he addressed to the Minister of Health in the House of Commons on the subject of hospitals, and the clear and emphatic reply given by Dr. Addison to the effect that there was no intention whatever to take over the voluntary hospitals or to set up State hospitals. But Mr. Humphrey, of Bradford, mentioned a municipal hospital, and that raised a different point. It might be that the Minister of Health was not going to set up competing hospitals, but could the Minister prevent a municipality, perhaps governed by an extremely Socialistic body, doing so? He would like to hear from Sir Robert Morant as to that.

**Mr. Woodcock** (Bradford) said that the sums which Sir Arthur Stanley had mentioned in his opening remarks would not be adequate for the upkeep of the hospitals, even with the existing number of beds. And one reason for the urgency of some new financial scheme was not only that the hospitals might be able to continue as they had been doing, but that they might cope more adequately with their waiting-lists, which were often big.

**Mr. Woods** (Chairman of Subscriptions Committee, Royal Victoria Infirmary, Newcastle), thought that the suggestion of Sir Arthur Stanley for subscriptions of a shilling and sixpence weekly was on the "high" side. It was desirable to extend the area of contribution. For the last fifteen years his hospital had received a considerable sum from the working classes of Northumberland and Durham, but not in one instance had it been at the rate of 1*s.* or even 6*d.* a week, and the working men of those counties were known to be exceedingly generous. It would be much better to appeal to working people for a penny or twopence a week than for a shilling or sixpence; and, by the way, it was part of the psychology of getting to ask for small sums frequently. A man would not promise to give four or five shillings a year when he would give a penny a week, which was the same thing. The more these men were told of the good work the hospitals were doing, the more readily they would respond.

**Sir Arthur Stanley** said that he wished to make it perfectly clear that the figures of 1*s.* and 6*d.* which he had mentioned at the beginning were purely hypothetical. It would be the same thing if, instead of a million people giving 1*s.*, twelve million people gave a 1*d.*, and even then it would only mean 30 per cent. of the population. With regard to the question raised by Mr. Lyle as to the competence of a municipality to build a hospital, there would surely be some Government department which would have power to prevent the borrowing of money, involving an increase of rates, to build a hospital, when it could be shown that it was possible to support the existing hospital by voluntary means. He thought that the best way he could answer the point about London was to say that one of the first bodies they wanted to work in harmony with was King Edward's Fund. (Hear, hear.) At the present moment King Edward's Fund in London was, at the request of the Red Cross, distributing and allocating a sum of £250,000 for the London Hospitals. If they could work together like that now, he did not see any difficulty in working together in the future. The principal object of the Red Cross and St. John's was to render first aid, and he thought the Provinces were really in greater need of first aid than London. The second branch of their work was home nursing, and perhaps the London hospitals would come in under that. (Laughter.) The Conference had given those responsible for the scheme a great deal of encouragement, and he was fully confident that it could be made a great success.

The resolution was then put and carried unanimously.

**Mr. Humphries** (Bradford) withdrew a motion he had handed in on the lines of his earlier speech, and the Conference broke up after a vote of thanks to the Chairman, moved by Sir Henry Burdett.

